# NMR Relaxometry Accessing the Relaxation Spectrum in Molecular Glass Formers

**DOI:** 10.3390/ijms23095118

**Published:** 2022-05-04

**Authors:** Manuel Becher, Anne Lichtinger, Rafael Minikejew, Michael Vogel, Ernst A. Rössler

**Affiliations:** 1 Nordbayerisches NMR Zentrum, Universität Bayreuth, 95440 Bayreuth, Germany; mbecher@nmr.physik.tu-darmstadt.de (M.B.); annelichtinger@gmx.de (A.L.); raf91@web.de (R.M.); 2 Institut für Physik Kondensierter Materie, Technische Universität Darmstadt, 64289 Darmstadt, Germany; michael.vogel@physik.tu-darmstadt.de

**Keywords:** molecular, ionic and confined liquids, glass transition, nuclear magnetic resonance relaxometry, dielectric spectroscopy

## Abstract

It is a longstanding question whether universality or specificity characterize the molecular dynamics underlying the glass transition of liquids. In particular, there is an ongoing debate to what degree the shape of dynamical susceptibilities is common to various molecular glass formers. Traditionally, results from dielectric spectroscopy and light scattering have dominated the discussion. Here, we show that nuclear magnetic resonance (NMR), primarily field-cycling relaxometry, has evolved into a valuable method, which provides access to both translational and rotational motions, depending on the probe nucleus. A comparison of ^1^H NMR results indicates that translation is more retarded with respect to rotation for liquids with fully established hydrogen-bond networks; however, the effect is not related to the slow Debye process of, for example, monohydroxy alcohols. As for the reorientation dynamics, the NMR susceptibilities of the structural (α) relaxation usually resemble those of light scattering, while the dielectric spectra of especially polar liquids have a different broadening, likely due to contributions from cross correlations between different molecules. Moreover, NMR relaxometry confirms that the excess wing on the high-frequency flank of the α-process is a generic relaxation feature of liquids approaching the glass transition. However, the relevance of this feature generally differs between various methods, possibly because of their different sensitivities to small-amplitude motions. As a major advantage, NMR is isotope specific; hence, it enables selective studies on a particular molecular entity or a particular component of a liquid mixture. Exploiting these possibilities, we show that the characteristic Cole–Davidson shape of the α-relaxation is retained in various ionic liquids and salt solutions, but the width parameter may differ for the components. In contrast, the low-frequency flank of the α-relaxation can be notably broadened for liquids in nanoscopic confinements. This effect also occurs in liquid mixtures with a prominent dynamical disparity in their components.

## 1. Introduction

In recent decades, the evolution of the dynamic susceptibility of molecular liquids undergoing the glass transition has been extensively studied using many experimental techniques, most notably dielectric spectroscopy (DS) [1,2,3,4], depolarized light scattering (DLS) [4,5,6,7], the optical Kerr effect (OKE) [8,9], and neutron scattering (NS) [10]. While NS probes density fluctuations, the former methods monitor reorientational dynamics. Upon (super-)cooling, a spectral gap opens between the microscopic dynamics in the sub-picosecond regime and the structural or α-relaxation shifts from the picoseconds time scale close to the boiling point to some hundreds of seconds around the glass transition temperature *T*_g_. Starting from the boiling point, with a more or less exponential correlation function, a stretched exponential long-time α decay emerges, usually already above the melting point. The stretching of the α-relaxation virtually does not change upon cooling when probed by, for example, DLS [4]. A weakly temperature-dependent partial loss is caused by fast dynamics. Together, two-step correlation functions constitute what is often called “glassy dynamics” [11,12,13]. Correspondingly, in the frequency domain, an asymmetrically broadened α-relaxation peak is observed at low frequencies in addition to a “microscopic peak”.

The spectral gap between the microscopic dynamics and the α-relaxation is filled with mostly distinctive relaxation contributions, usually referred to as secondary processes. These are more difficult to access experimentally compared to the main structural relaxation; in particular, they are difficult to observe in the time domain. Moreover, these secondary relaxations cover a broad time/frequency range extending from the microscopic dynamics in the sub-picosecond range to that of the α-relaxation, and it is often difficult to define a characteristic time scale. In other words, in contrast to crystals, there is a continuum of overlapping relaxation features faster than the α-relaxation, which may cover a spectral range from picoseconds to seconds.

The secondary relaxations may involve an excess wing contribution and/or a β-relaxation [1,14,15,16,17]. The former manifests itself as a further high-frequency flank of the main relaxation peak, described by a power-law-like “excess wing” [18]; the latter refers to a mostly resolved second relaxation peak. Usually, both relaxation features emerge only at temperatures near *T*_g_, and in most cases they are difficult to disentangle from the main relaxation. As such, they interfere with a straightforward determination of the extent of the α-relaxation stretching itself. In contrast, below *T*_g_, the structural relaxation no longer occurs on relevant time scales, and a distinct secondary relaxation peak can be observed, which is usually referred to as the Johari–Goldstein β-process [14], and/or a mostly isolated excess wing shows up as a power-law spectrum [16].

The dielectric spectra of liquids displaying no discernible β-relaxation, so-called type A glass formers [1], were analysed by Nagel and co-workers [19], who suggested a scaling for the α-relaxation and excess wing; however, this works only approximately [20,21,22]. Focusing on a series of such systems, Körber et al. recently re-analysed the relaxation spectra close to *T*_g_ by taking the power-law contribution of the excess wing explicitly into account [23]. In particular, DS results were compared with those obtained by NMR and DLS. The analysis showed that NMR and PCS yielded a similar relaxation stretching, which varied only weakly among various liquids and resembled that found in DS, provided that the liquid was nonpolar. Explicitly, Kohlrausch–Williams–Watts (KWW) stretching parameters close to $\beta_{K}=0.6$ were reported [23]. For polar liquids, the dielectric relaxation spectra are narrower [24,25], which is reflected in higher DS stretching parameters compared to those reported from NMR and PCS. Pabst et al. investigated PCS spectra in more detail and stated that “the light scattering spectra of different systems, e.g., hydrogen bonding and van der Waals liquids but also ionic systems, almost perfectly superimpose and show a generic line shape of the structural relaxation, approximately following a high frequency power law $\omega^{-1/2}$” [26]. If such a generic NMR and DLS stretching of the α-relaxation can be confirmed, it is important to understand the origin of the DS finding, which is that $\beta_{K}$ varies significantly with temperature and among different liquids. Pabst et al. suggested that cross-correlation effects may be responsible for the particularities observed in dielectric spectra [27,28]. This has also been suggested by recent molecular dynamics (MD) simulations of polymer-plasticizer systems [29]. Experimentally, significant differences between NMR and DS results for binary glass formers point in the same direction [30]. However, the role of cross-correlation effects has not received much attention in experimental studies thus far [31].

A priori, it is not clear whether or not the rotational degrees of freedom of an anisotropic, non-rigid molecule all couple to the same extent as the structural relaxation, the latter being defined by shear relaxation, for example. There exists a vast amount of literature documenting anisotropic reorientation in low-viscosity liquids, which is more or less controlled by the corresponding moments of inertia; however, the extent of the anisotropy is often small [32,33,34]. Moreover, given a non-rigid molecule, one would expect a temperature-dependent distribution of conformers, which could differ with respect to their dynamical behaviours. Hence, the observed temperature dependence may deviate from that of the structural (shear) relaxation time *τ*_α_(*T*). Different from DS and DLS, NMR allows one to probe the dynamics of different sites within a molecule—in particular, when using the isotope selectivity of the method and suitable labelling strategies. Recently, from surveys of extensive ^2^H NMR relaxation data sets, stretching parameters in the range of 0.39 < *β*_K_ < 0.70 were reported well above *T*_g_ [35]. This implies a much wider variation of the α-relaxation shape than suggested by the above-mentioned studies. Yet, indications were found that these site-specific variations tend to vanish upon further cooling. In other words, non-rigid molecules may start to reorient more or less isotropically as a rigid entity when the highly viscous regime close to *T*_g_ is approached; this feature was recently confirmed by experiments as well as an MD simulation in the case of glycerol [36]. The suppression of anisotropic reorientation in the highly viscous state has since been discussed in [37,38].

Most information collected on the dynamics close to *T*_g_ still originates from DS, and the results are often considered as a de facto standard for discussing the (reorientational) dynamics of glass formers, in particular, when secondary relaxations are considered. Only recently, these relaxation features have been accessed by DLS [27,28] and OKE [39], and only few studies have attempted a direct comparison of the different techniques [4,27,40]. Moreover, field-cycling (FC) NMR has entered use and provided valuable insights [41,42,43]. Conventional high-field (HF) relaxation studies monitor the spin-lattice relaxation rate *R*_1_ at a single or very few (Larmor) frequencies determined by the employed cryo-magnet. Thus, conventional NMR relaxometry suffers from a notorious lack of sufficient dispersion data, rendering the analysis model dependent. The situation has improved since commercial [42,43,44,45] and home-built FC relaxometers have become available [46,47,48,49]. In FC relaxometry, switchable electromagnets are used to measure the frequency dependence of $R_{1}\left(\omega \right)$, typically in the range of typically 10 kHz–30 MHz [42,50]. Using home-built machines, even a range of 100 Hz–40 MHz can routinely be accessed [46,47]. However, compared to DS, the frequency range covered is still narrow. As will be demonstrated below, it is often possible to overcome this limitation. Then, NMR relaxometry may provide important information not offered by DS or DLS. Most FC studies employ the ^1^H nucleus; however, ^2^H, ^7^Li, ^19^F, and ^31^P NMR studies are becoming feasible, too [48,49,51,52,53]. To better understand the α-relaxation stretching and the manifestation of secondary relaxation processes in viscous liquids as detected by the different spectroscopic techniques, it is important to carefully compare the evolution of the respective relaxation spectra and clarify what specific aspect of the underlying molecular dynamics the various experimental techniques probe.

NMR spin-lattice relaxation is unique in several respects. ^2^H and ^31^P relaxation studies (the latter at sufficiently high fields, see below) provide access to *single-particle* reorientational correlation functions, where the term ‘single particle’ refers to the fact that the autocorrelation of the molecular orientation at two times is probed. In contrast, for other nuclei, such as ^1^H, multi-particle interactions (such as magnetic dipole–dipole interactions) prevail and *collective* aspects of the relaxation are probed, a situation also encountered in DS [31]. Here, collective correlation refers to the orientational correlation between one molecule and another molecule. Moreover, NMR relaxation does not suffer from the two major drawbacks of DS approaches. It provides straightforward access not only to polar systems but also non-polar systems, which allows one to significantly extend the spectrum of the studied systems, and, importantly, parasitic conductivity contributions do not interfere.

Other NMR techniques, such as line-shape analysis and stimulated-echo experiments, have provided important information regarding the mechanism of molecular reorientations underlying the α- and β-processes [3,54]. For example, it was reported that the α-relaxation involves a mixture of many small-angle (2°–3°) and few large-angle (30°–50°) jumps, where the latter are more relevant for the overall loss of orientational correlation. Regarding the Johari–Goldstein β-process in neat glass formers, NMR techniques have disclosed the highly hindered reorientations of essentially all molecules (*T* < *T*_g_) [55,56,57,58]. No indications of island of mobility were found in these studies. Rather, wobbling-in- or on-a-cone models captured the salient features of the experimental observations [57,58]. On the other hand, FC NMR relaxometry—similar to DS—offers quantitative access to the detailed shape of the dynamical susceptibilities and spectral densities of glass-forming liquids or other complex relaxation processes in condensed matter [59,60]. This technique allows one to cover several decades in amplitude, revealing information concerning not only the α-relaxation but also the relaxation spectrum of secondary processes, such as the excess wing and β-relaxation.

A main goal of the present contribution is to compare the dynamic susceptibilities of glass-forming liquids obtained from DS, DLS, and NMR studies. In such an approach, it is necessary to consider that different ranks (*l*) of the reorientational correlation function may be probed. Explicitly, the correlation function of the first-rank (*l* = 1) Legendre polynomial can be probed by DS, whereas that of the second-rank (*l* = 2) can be observed with DLS and NMR [31,54]. Different motional models yield *l*-dependent (relative) relaxation strengths of the α- and β-processes, for instance. Hence, once again, it is of major interest to quantitatively compare the susceptibility spectra probed by different spectroscopic methods. Here, we will present results for (simple) molecular liquids, monohydroxy alcohols, salt solutions, and ionic liquids. Moreover, the effects of nanoscale confinements on liquid dynamics will be addressed. In doing so, NMR relaxation data for ^1^H, ^2^H, ^7^Li, and ^31^P nuclei will be compiled.

## 2. Spin-Lattice Relaxation and Dynamic Susceptibility

The spin-lattice relaxation rate $R_{1}\left(\omega \right)$ is linked to the spectral density *J*(*ω*) [≡ *J*_l=2_(*ω*)] via a Bloembergen–Purcell–Pound (BPP)-type equation [61]. Referring to, for example, a homonuclear dipolar (^1^H) or a quadrupolar (^2^H) relaxation mechanism, the relaxation rate can be re-formulated in the susceptibility representation as [62]:

(1) $$\omega R_{1}\left(\omega \right)=K\left[\omega J\left(\omega \right)+4\omega J\left(2\omega \right)\right]=K\left[\chi^{\prime \prime }\left(\omega \right)+2\chi^{\prime \prime }\left(2\omega \right)\right]\equiv \chi_{NMR}^{\prime \prime }\left(\omega \right)\;(\mathrm{dipolar}\;\mathrm{or}\;\mathrm{quadrupolar}\;\mathrm{interaction})\;$$

In words, by virtue of the fluctuation-dissipation theorem, the $R_{1}\left(\omega \right)$ data are transformed to their susceptibility representation $\chi_{NMR}^{\prime \prime }\left(\omega \right)$ such that the NMR relaxation results can directly be compared to those from DS or PCS, for example. The pre-factor *K* characterizes the strength of the relevant spin interaction, which is usually taken as being temperature independent. It should be noted that “true” single-frequency susceptibilities are actually not obtained, because two susceptibility functions appear in Equation (1), slightly broadening $\chi_{NMR}^{\prime \prime }\left(\omega \right)$ when compared to other methods, such as DS or DLS, which has to be considered in the fitting routine. Another result is that the peak frequency satisfies the condition $\omega_{peak}\tau_{peak}\approx 0.62$ instead of 1. Therefore, when comparing NMR susceptibilities to DS or DLS on a reduced frequency scale $\omega \tau_{peak}$, the factor 0.62 has to be accounted for. In the case of FC ^31^P NMR studies of organic liquids, the spin-lattice relaxation is usually determined by the heteronuclear dipolar interaction between the phosphorus and proton spins and is described by a relation similar to Equation (1) [33,53]. In contrast, the ^31^P relaxation at high magnetic fields is dominated by the fluctuation of the chemical shift anisotropy (CSA), with the particularity that the coupling constant *K* depends on ω^2^ [33,61], and only a single susceptibility function appears in the BPP treatment; the susceptibility representation is given by [53]:

(2) $$R_{1}\left(\omega \right)/\omega \equiv \chi_{NMR}^{\prime \prime }\left(\omega \right)\;\;\;\;\;(\mathrm{CSA}\;\mathrm{interaction})$$

The ^2^H (*I* = 1) quadrupolar relaxation in organic molecules results from the fluctuating interaction of the deuteron with the electric field gradient (EFG) produced by the chemical bond at the nuclear site; hence, it probes bond reorientation. Similarly, in the case of the CSA interaction, single-particle reorientation is probed. Under such circumstances, the spectral density reflects the Fourier transform of the corresponding (*l* = 2) rotational correlation function. Furthermore, the respective interaction tensor is largely determined by the chemical bonds of the molecule and, in particular, the coupling constant *K* is well defined and temperature independent in the first approximation. In ^7^Li NMR, the situation is more complicated [63,64]. Similar to ^2^H NMR, the spin-lattice relaxation of this quadrupolar nucleus (*I* = 3/2) results from the fluctuation of the EFG at the nuclear site. However, the EFG, unlike in ^2^H NMR, is not determined by a single covalent bond, but by the charge distribution around the lithium ion in ^7^Li NMR, e.g., by the structure of the hydration shell in the case of LiCl solutions. Hence, ^7^Li NMR relaxation probes collective dynamics, which involve rotational and translational motions. Moreover, the parameters of the quadrupolar interaction tensors, including the coupling constant *K*, are subject to broad and a priori unknown distributions.

For homonuclear dipolarly coupled spins (^1^H and ^31^P at low fields), there exists an important particularity. The spin-lattice relaxation involves an intra- and an intermolecular pathway. The intramolecular part reflects the reorientational dynamics of the molecules, which are usually considered as rigid, whereas the intermolecular part contains two contributions: translational and reorientational. As a heuristic but tractable approach, we assume that the spectral shape of $R_{1,\mathrm{inter}}^{\mathrm{rot}}\left(\omega \right)$ is similar to that of $R_{1,\mathrm{intra}}^{}\left(\omega \right)$ [50]. Hence, the total relaxation rate can be described by a sum of a rotational and a translational contribution, specifically:

(3) $$R_{1}\left(\omega \right)≅R_{1,\mathrm{intra}}^{}\left(\omega \right)+R_{1,\mathrm{inter}}^{\mathrm{rot}}\left(\omega \right)+R_{1,\mathrm{inter}}^{\mathrm{trans}}\left(\omega \right)≅R_{1}^{\mathrm{rot}}(\omega )+R_{1,\mathrm{inter}}^{\mathrm{trans}}(\omega )$$

Here, $R_{1}^{\mathrm{rot}}\left(\omega \right)$ will be described by spectral densities known from DS or PCS studies, for example, a Cole–Davidson (CD) function [65], or even two CD functions when the excess wing needs to be considered in addition to the α-relaxation [23,36]; $R_{1,\mathrm{inter}}^{\mathrm{trans}}(\omega )$ will be analyzed in the framework of the force-free hard sphere (FFHS) model (see below).

Independent of any microscopic details of the translational motion, the molecules in a liquid undergo free diffusion at sufficiently long times, implying a mean square displacement that is linear in time, $\langle \Delta r^{2}\rangle =6D\;t$. In this limit, the translational correlation function displays a power-law long-time behaviour, explicitly $C_{\mathrm{trans}}\left(t\right)\propto t^{-3/2}$, corresponding to a low-frequency square root *R_1_* dispersion [66].

(4) $$R_{1,\mathrm{inter}}(\omega )=R_{1,\mathrm{inter}}\left(0\right)-\frac{B}{D^{3/2}}\cdot \sqrt{\omega }\;+.\;.\;.$$

with $B={\left(\frac{\mu_{0}}{4\pi }\hbar \gamma^{2}\right)}^{2}\left(\frac{1+4\sqrt{2}}{30}\right)\;\pi \;n\;$.

Here, *n* denotes the spin density and *γ* represents the gyromagnetic ratio. As the power-law decay of *C*_trans_(*t*) always wins over the (stretched) exponential correlation function of *C*_rot_(*t*) at sufficiently long times, Equation (4) also provides a universal low-frequency dispersion law for the total relaxation rate $R_{1}\left(\omega \right)$ and enables the determination of the self-diffusion coefficient *D* in pure liquids [49,52,67,68].

The full relaxation spectrum of the translational motion is usually well captured by the FFHS model. In this model, a distance of closest approach *d* (twice the hard sphere’s radius) is introduced, and the (normalized) spectral density *J*_trans_ (*ω*) takes the following form [69,70,71,72]:

(5) $$J_{\mathrm{trans}}\left(\omega \right)=\;\frac{54}{\pi }\;{\int_{0}^{\infty }\frac{u^{2}}{81+9u^{2}-2u^{4}+u^{6}}\frac{u^{2}\tau_{\mathrm{trans}}}{u^{4}+{\left(\omega \tau_{\mathrm{trans}}\right)}^{2}}du}^{\;}$$

The corresponding time constant *τ*_trans_ is related to the self-diffusion coefficient by:

(6) $$\tau_{\mathrm{trans}}=\frac{d^{2}}{2D}$$

The related coupling constant *K*_trans_ is given by:

(7) $$K_{\mathrm{trans}}={\left(\frac{\mu_{0}}{4\pi }\right)}^{2}\frac{8\pi }{15}\frac{n}{d^{3}}I\left(I+1\right)\hbar^{2}\gamma^{4}$$

Fitting the low-frequency part of $\chi_{NMR}^{\prime \prime }\left(\omega \right)$ with Equation (1) and employing Equations (5) and (7) provide the parameter *d*. It should be noted that the suitability of the FFHS model was recently confirmed by MD simulations [36,71].

Thus, FC ^1^H NMR relaxometry allows us to determine both *D* and *τ*_rot_. Assuming the Stokes–Einstein–Debye (SED) relation, their product is a measure of the hydrodynamic radius *R*_H_ [73,74].

(8) $$D\tau_{\mathrm{rot}}=\frac{2}{9}R_{H}^{2}$$

It should be noted that Equation (8) assumes rotational diffusion, i.e., small-step reorientation. In general, estimating the absolute values of *R*_H_ from $D\tau_{\mathrm{rot}}\;$depends on the assumed motional model. In the frame of the FFHS model, which does not assume the validity of the SED relation, *τ*_trans_ is linked to the distance *d* Equation (6). When defining the ratio *r* = *τ*_trans_/*τ*_rot_ as a measure of the separation of translation and rotation in a liquid, we obtain the following [36,50,74]:

(9) $$r=\frac{\tau_{\mathrm{trans}}}{\tau_{rot}}=\frac{d^{2}}{2D\tau_{rot}}$$

When we further assume that the SED relation is valid and *d* = 2*R*_H_ holds, we arrive at *r* = 9 [50,61]. In other words, within the hydrodynamic approach, the spectral separation of translational and rotational relaxation contributions is independent of the molecular size and rather small; hence, it is difficult to resolve. However, as will be discussed below, the ratio *r* is much larger for some liquids, indicating a failure of the SED relation, which goes along with unphysically small hydrodynamic radii as RH2=9 d24r holds [36].

For liquids obeying frequency-temperature superposition (FTS), the dynamic susceptibility can be expressed by χ’’(ωτ), where *τ* is a characteristic time constant, for instance *τ*_rot_ [4,11,62,75]. This leads to the possibility of constructing master curves by shifting the individual $\chi ’’\left(\omega \right)$ data collected at different temperatures solely along the *ω* axis until they overlap. Assuming that the NMR coupling constant *K* does not significantly change with temperature, master curves are obtained for ${\chi^{\prime \prime }}_{NMR}=\omega R_{1}\left(\omega \right)$ [62]. Vice versa, it suffices to measure χ’’ at a single frequency as a function of *τ*(*T*). Here, one has to keep in mind that the obtained master curve χ’’(ωτ)characterizes the spectral shape of the susceptibility of a typical glass-forming liquid at the lowest temperature used in the construction of the master curve. Below, we will show that the application of FTS is an important tool to effectively extend the still narrow frequency window of FC NMR.

## 3. Experimental Results

### 3.1. Simple Liquids

Some intriguing results obtained by FC NMR relaxometry in recent years include the determination of the shape of the α-relaxation and the identification of an excess wing on its high-frequency flank as a generally occurring spectral feature observed at temperatures close to *T*_g_. The latter feature is well known from DS [2], but is still an experimental challenge when applying DLS, though indications have been reported [4,28,76]. Another important result concerns the fact that both the translational diffusion coefficient *D*(*T*) and the rotational correlation time *τ*_rot_(*T*) can be determined from the ^1^H NMR susceptibility $\chi_{NMR}^{\prime \prime }\left(\omega \right)$; thus, the SED relation can be tested in a single experiment, revealing an SED breakdown, in particular, in hydrogen-bonded liquids (see below).

**Figure 1:**
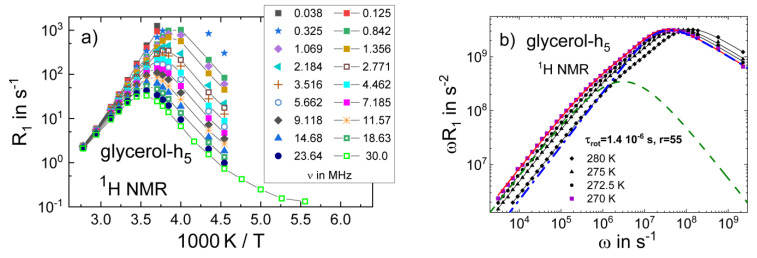
(**a**) ^1^H relaxation rate *R*_1_ of partially deuterated glycerol-h_5_ at exemplary Larmor frequencies (*ω*/2π) as a function of reciprocal temperature [35]; a spectral range typical of commercial FC relaxometers is covered. The lines are guides to the eye. (**b**) Susceptibility spectrum *χ*″(*ω*) = *ωR*_1_(*ω*) as obtained from a home-built instrument together with high-field data. The red solid line represents a fit by a sum of two sub-spectra referring to the translational (dashed line) and rotational (dashed-dotted line) relaxation contributions at 270 K, yielding the indicated parameters (see text for details).

In Figure 1a, we present the results of a ^1^H FC study of glycerol-h_5_ [35]. The spin-lattice relaxation rate *R*_1_(*T*) at different frequencies is plotted as a function of reciprocal temperature, as is typical of conventional high-field (HF) studies. Data in the frequency range 20 kHz–30 MHz are included, as available from commercial relaxometers. As expected from Equation (1), the amplitude and position of the *R*_1_ maxima depend on the frequency. A larger spectral window is available when recourse is taken to a home-built relaxometer [46,47], as was found in studies of polymer dynamics, for example, in [77]. This possibility is demonstrated for glycerol-h_5_ in Figure 1b, where the susceptibility spectra *χ*″(*ω*) = *ωR*_1_(*ω*) at 270–280 K are shown. Applying earth field compensation, frequencies down to 200 Hz are accessible. In addition, data from conventional HF experiments at 92 MHz and 360 MHz are included. In this way, six decades in frequency are covered, and, as will be described below, all relevant parameters of the dynamics can be extracted from a single relaxation spectrum. However, such broad-band FC NMR spectra are not yet routinely available, and in many cases, one has to make do with the 3–4 decades in frequency provided by commercial relaxometers. Given such a limited spectral range, a clear picture of the relaxation spectrum cannot emerge. Here, nature in terms of FTS may help, as will be demonstrated next.

**Figure 2:**
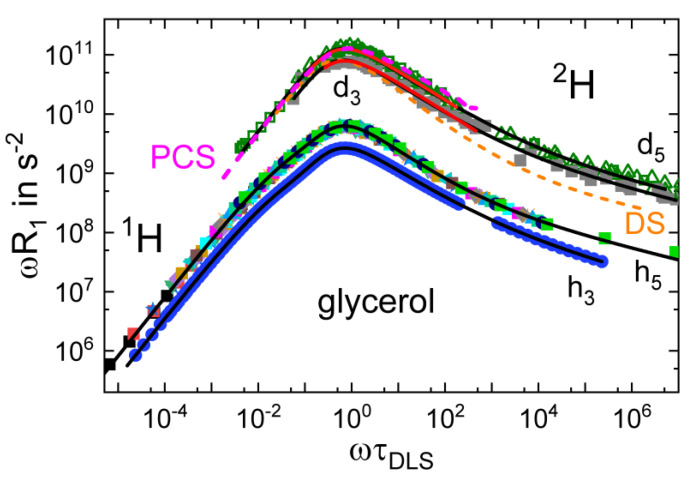
Susceptibility master curves as a function of $\omega \tau_{\mathrm{DLS}}\left(T\right)$ (τ _DLS_ data from ref. [28]). The plot includes ^1^H FC data for glycerol-h_3_ (blue closed circles) [36] and glycerol-h_5_ (multi-coloured data) [36], and high-field ^2^H spin-lattice relaxation data for glycerol-d_3_ (dark green) and glycerol-d_5_ (grey) recorded at 55 MHz (squares) and 40 MHz (triangles) [78]. The master curves include results at temperatures from 180 K to 360 K, the dielectric spectrum at 288 K (orange dashed line) [79], and the PCS spectrum at 220K (magenta dashed line). Black lines represent fits with a model featuring translational and reorientational contributions (see text) for ^2^H data with reorientational contribution only. Red lines show a fit of the maximum region with a single CD function, yielding a stretching parameter *β*_CD_ ≈0.47±0.03.

Figure 2 shows the data from Figure 1a after (i) mapping $R_{1}\left(T\right)$ to $\omega R_{1}\left(\tau_{\mathrm{DLS}}\right)$ using correlation times $\tau_{\mathrm{DLS}}$(*T*) from DLS [28] and (ii) rescaling the $\tau_{\mathrm{DLS}}$ axis by the Larmor frequency ω. A perfect overlap of all data is obtained without applying any data shifting, thus demonstrating that FTS is valid and that the time scale of the relaxation process probed by NMR is at least proportional to that monitored by DLS. In fact, both time scales are absolutely the same because the relaxation maximum in Figure 2 occurs at *ωτ*_DLS_ ≈ 1. The master curve covers more than 12 decades in frequency and its shape characterizes the susceptibility at the lowest temperature included in its construction (a temperature close to *T_g_*). Clearly, in addition to the α-relaxation peak, a high-frequency power law contribution with a low apparent exponent—the excess wing—is revealed. On the low-frequency flank, the spectrum displays a weak shoulder, which results from the intermolecular dipolar relaxation contributions probing molecular translation [52]. As discussed in Section 2, intermolecular ^1^H relaxation caused by translational dynamics dominates at low frequencies and leads to a separate weak relaxation peak, which shows up as a low-frequency shoulder in the overall spectrum.

In Figure 2, we added the FC ^1^H data for glycerol-h_3_ [36]. Very similar spectral shapes are present for the compounds with different isotope-labelling schemes. The difference in amplitudes is explained by the different strength of the dipolar couplings among the protons in the molecule. Moreover, we included HF ^2^H *R*_1_ data for both partially deuterated glycerol compounds after rescaling them by ω [78]. The ^2^H data and the ^1^H data have the same spectral shape near the peak and at higher frequencies. Importantly, although ^2^H *R*_1_ is only measured at a single frequency, the validity of FTS allows us to reveal the full susceptibility, including an excess wing, which resembles that of the ^1^H FC susceptibility. Apparently, the rotational parts of all ^1^H and ^2^H relaxation spectra are essentially identical. In particular, there are no differences between glycerol-h_3_ and glycerol-h_5_, indicating that the non-rigid molecule glycerol virtually reorients as a rigid entity and providing no evidence for anisotropic reorientation [78]. Since ^2^H relaxation solely probes reorientational dynamics, these data do not show a translation-caused shoulder at low frequencies. The translationally caused relaxation is actually best recognized in the time domain when plotting the NMR correlation function obtained from the susceptibility master curves; more precisely, the corresponding spectral densities by Fourier transformation, which will be discussed below.

To describe the reorientational relaxation peak and the excess wing, we applied a fit involving a weighted sum of two CD functions. Alternatively, a sum of a Kohlrausch and a CD function is also suitable [23]. The intermolecular ^1^H low-frequency relaxation contribution is interpolated by the FFHS model, which describes dipolarly coupled spins undergoing translational diffusion (see Section 2). The fit yields *τ*_rot_(*T*) (see Figure 1b) and *τ*_trans_(*T*), which are related by a virtually temperature-independent ratio, $\tau_{\mathrm{trans}}/\tau_{\mathrm{rot}}=r\approx 55$. This value of *r* is much larger than the ratio *r* = 9 expected from the SED relation. A similar fit describes the ^1^H relaxation data of glycerol-h_3_ well. To allow for a quantitative comparison with analyses that do not consider the excess wing, we also fitted only the peak regions with a single CD function (red line in Figure 2). For the ^1^H relaxation data, the fit yields *β*_CD_ = 0.45 (*β*_K_ = 0.60) for glycerol-h_3_ and *β*_CD_ = 0.47 (*β*_K_ = 0.63) for glycerol-h_5_. For glycerol-d_3_ and glycerol-d_5_, the ^2^H relaxation data yield a similar value of *β*_CD_ = 0.49, confirming the assumption underlying Equation (3). Finally, we included the DS and DLS spectra of glycerol in Figure 2. Clearly, the dielectric relaxation peak is narrower; in particular, the amplitude of the excess wing is smaller. In the case of glycerol, one can describe the full dielectric spectrum using the stretching parameter consistently found by NMR and DLS and a weaker excess wing contribution [80]. However, in light of recent investigations suppressing a possible collective dielectric relaxation by dilution experiments [28], it may also be possible that the dielectric α-peak itself is narrower due to a significant contribution from collective correlation effects. As mentioned earlier, highly polar liquids, such as glycerol, generally display higher dielectric stretching parameters than less polar liquids [24,25].

**Figure 3:**
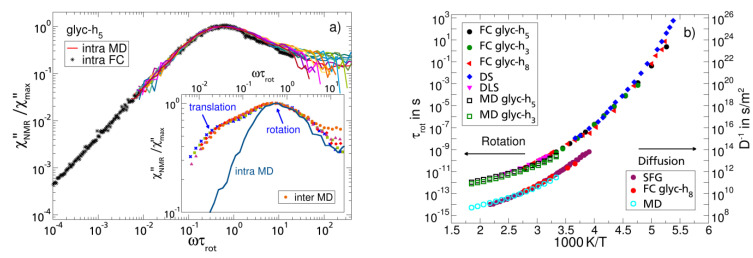
(**a**) Master curves of the intramolecular FC susceptibility *χ*″_intra_ of glycerol-h_5_ as a function of the reduced frequency *ωτ*_rot_ [72] (black circles) together with the results from MD simulations (coloured lines) [36]. Data are scaled by the maximum values *χ*″_max_. Inset: simulation results for the intra- and intermolecular relaxation contributions on a reduced frequency scale. The bimodal structure, reflecting translational and rotational dynamics, is well recognized in the intermolecular part. (**b**) Rotational correlation times *τ*_rot_ of glycerol as obtained from NMR [36] and other techniques (DS [1] (blue diamonds), DLS [81] (magenta triangles), and MD simulations [36]) together with inverse self-diffusion coefficients *D*^−1^ from static field gradient diffusometry [82] (SFG, purple circles), FC relaxometry on glycerol-h_8_ [50], and MD simulations [36].

Given the fit parameters *τ*_trans_(*T*) and the translational coupling constant *K*_trans_, one can calculate the diffusion coefficients *D*(*T*) using Equations (6) and (7) [67]. The results agree well with those obtained from static field gradient NMR experiments (see Figure 3b). Alternatively, *D*(*T*) can be directly determined from the low-frequency square root dispersion law Equation (4). This possibility will be demonstrated below for the case of mono-alcohols (Section 3.4).

In another study, glycerol was also investigated by atomistic MD simulations to investigate the rotational and translational dynamics and their relations to the ^1^H relaxation [36]. Good agreement between the experiment and simulation was found. For example, the simulation data reproduced the experimental intramolecular ^1^H relaxation contribution (Figure 3a), which was determined in an independent isotope dilution experiment [72]. Such experiments allow one to single out the intra- and intermolecular dipolar relaxation contributions. The simulation data also confirm the unusually large separation of the time scales of the rotational and translational motions, which is at variance with the SED relation (see the inset of Figure 3a). The bimodal (translational and rotational) spectral shape of the intermolecular relaxation contribution is evident, whereas the intramolecular rotational contribution displays a “CD-like” spectrum. The time constants from the experiment and simulation are compared in Figure 3b, revealing high similarity.

**Figure 4:**
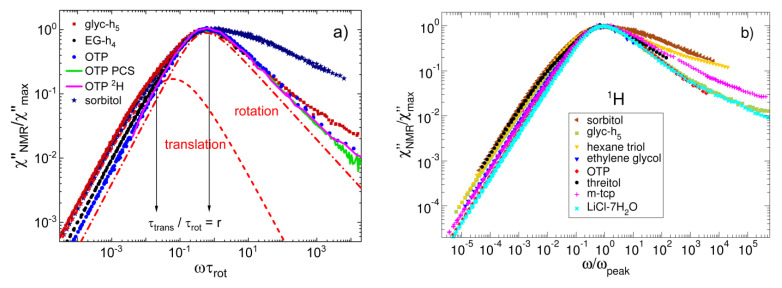
(**a**) NMR susceptibility master curves of o-terphenyl (OTP), ethylene glycol-h_4_ (EG-h_4_) [84], glycerol-h_5_ (glyc-h_5_), and sorbitol scaled to overlap in the peak region [85]; the *r* value characterizing the separation of translational and rotational time constants increases from 10 (o-terphenyl) to 26 (ethylene glycol) to 54 (glycerol and sorbitol). In the case of o-terphenyl, the susceptibilities obtained from ^2^H relaxation [86] and from DLS [87] are included. (**b**) FC susceptibility master curves of the eight liquids studied so far.

The large separation of rotation and translational dynamics, i.e., the high *r* value, goes along with a failure of the SED relation and an unphysically low hydrodynamic radius *R*_H_ [74]. In the case of glycerol-h_5_, a value of *R*_H_ = 0.09 nm is found based on Equation (9) [74], which is much smaller than the van der Waals radius of 0.27 nm [83]. Likewise, an unreasonably small hydrodynamic radius was obtained from an SED analysis in the literature [73]. In order to unravel the origin of this phenomenon, we measured the FC ^1^H relaxation of molecules with different numbers *n*_OH_ of hydroxyl groups. The van der Waals liquid o-terphenyl (*n*_OH_ = 0) and ethylene glycol (*n*_OH_ = 2) had a smaller number of hydrogen bonds than glycerol (*n*_OH_ = 3), and this number further increased in the homologous series from glycerol to sorbitol (*n*_OH_ = 6). The FC ^1^H NMR master curves of some of these liquids are shown in Figure 4a. Independent of the magnitude of the electric dipole moment of the molecules, the master curves of all liquids show essentially the same rotational relaxation peak, while the excess wing contribution differs somewhat. However, the manifestation of the translational spectrum mediated by the dipolar intermolecular interaction was quite different. The value of *r* systematically decreases from glycerol (r ≈ 55), over ethylene glycol (*r* ≈ 26) [84], to o-terphenyl (*r* ≈ 10). On the other hand, a similarly high value of *r* ≈ 54 was found throughout the homologous series with *n*_OH_ = 3–6. This can be seen from the example of sorbitol, which showed a low-frequency shoulder similar to that of glycerol but a significantly different high-frequency behaviour due to a strong *β*-process. These findings imply that the separation of rotation and translation grows when the hydrogen-bond density increases, until a hydrogen-bond network is fully established for the case of glycerol [84]. A further rise to *n*_OH_ = 6 did not further increase the network connectivity and the time-scale separation. The conjecture that ratios of *r* > 9 are related to mostly established hydrogen bond networks is corroborated by the findings that diluting propylene glycol with a nonpolar solvent, i.e., destroying the hydrogen-bond network, reduces the *r* value from about 40 to 10 [74].

In Figure 4a, we added the NMR susceptibility $\chi_{NMR}^{\prime \prime }\left(\omega \right)\;$of o-terphenyl as obtained from HF (single frequency) ^2^H spin-lattice relaxation measurements [86]. As described above, taking the time constants from a DLS study [87], a susceptibility can be constructed from *R*_1_(T) data. The ^2^H susceptibility agrees with that from FC ^1^H relaxometry in the peak region; however, as expected, it does not show a low-frequency shoulder because the ^2^H relaxation is governed by the quadrupolar interaction, which is basically free of intermolecular contributions. Furthermore, the PCS susceptibility of o-terphenyl is included [87], again displaying almost perfect agreement with the NMR data. Comparing the results for glycerol, ethylene glycol, and o-terphenyl, some differences are observed with respect to the manifestation of the excess wing, indicating that the amplitude of this contribution is system-dependent. This is confirmed in Figure 4b, where we compare the FC ^1^H NMR susceptibilities of most of the glass-forming liquids studied so far. Clearly, the high-frequency flank is not universal. However, some of the differences may result from a hidden β-relaxation, which continuously separates from the α-relaxation upon cooling. For hexanetriol and the higher glycerol homologues, the existence of a β-relaxation was confirmed in the dynamic bulk and shear modulus [88] and dielectric spectra [89].

A main advantage of NMR is its isotope selectivity, which allows us to probe the site-specific dynamics. Above, this was demonstrated for glycerol compounds with different isotope labelling, revealing that the non-rigid molecule glycerol behaves essentially as a rigid entity in the viscous liquid. The question is whether this phenomenon is common to liquids approaching their glass transition. Rather than monitoring the relaxation of differently labelled molecules using a single probe nucleus, it is also possible to simultaneously study the relaxation of different molecular entities of the same molecule by using various probe nuclei, e.g., ^1^H and ^31^P in the case of m-tricresyl phosphate (m-TCP); however, FC ^31^P data are still difficult to collect routinely, and measurement times are very long [53].

**Figure 5:**
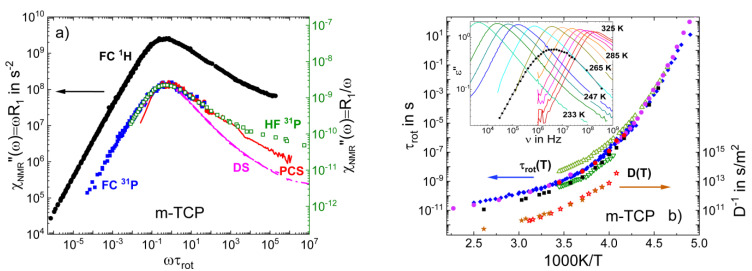
(**a**) NMR susceptibility master curves of m-tricresyl phosphate (m-TCP) determined from FC ^1^H relaxometry (black circles; constructed from data in the range 220 K–383 K), ^31^P NMR relaxometry (blue squares; data in the range 240 K–290 K), and high-field ^31^P NMR (green open squares) [90]. The latter are dominated by the CSA interaction and rescaled in amplitude (right ordinate). Added are the PCS (red line) [4]) and DS (magenta line) [93,94] susceptibilities close to *T*_g_. (**b**) Rotational correlation times of m-TCP as obtained from different techniques: DS—open green triangles [53]; high-field ^31^P NMR relaxation—blue squares [90]; FC ^31^P NMR—red circles [53]; FC ^1^H NMR—black squares [53]; and DLS—violet circles [92]. Reciprocal diffusion coefficient D^−1^(T) measured by FC relaxometry (open orange stars) [53] and by static field gradient (SFG) diffusometry (solid brown stars) [53]—right ordinate. Inset: dielectric spectra of m-TCP at high temperatures revealing a bimodal spectral shape; included is a FC ^1^H NMR spectrum at the corresponding temperature.

Figure 5a displays the FC susceptibility master curves of m-TCP obtained by FC ^1^H and FC ^31^P NMR relaxometry [53]. Additionally, HF ^31^P relaxation data are included [90,91], which extend the susceptibility spectrum to higher frequencies. In the HF study, ^31^P *R*_1_(T) was measured at three Larmor frequencies ω, and the susceptibility master curve resulted from transforming $\omega R_{1}\left(T\right)\;$to $\omega R_{1}\left(\tau_{\mathrm{DLS}}\right)$, taking $\tau_{\mathrm{DLS}}$(*T*) from DLS [92], and rescaling the $\tau_{\mathrm{DLS}}$ axis by ω. Thus, a master curve was again not enforced by shifting spectra measured at different temperatures for best overlap, but just results as a consequence of the applicability of FTS and the consistency of the NMR and DLS correlation times. The ^1^H and ^31^P NMR susceptibility master curves have very similar shapes, including the excess wing contribution. A fit of the relaxation peak yields *β*_CD_ = 0.41 for both nuclei, similar to the stretching found for glycerol, ethylene glycol, and o-terphenyl. We also added the DS [93,94] and DLS spectra [4] of m-TCP measured close to *T*_g_. Similar to glycerol, the NMR and DLS susceptibilities agree around the α-peak, whereas the dielectric spectrum is significantly narrower, as may be expected due to m-TCP being a polar liquid.

The NMR, DLS [92], and DS [53] rotational correlation times of m-TCP are displayed in Figure 5b. Three *τ*_rot_(*T*) traces are observed at high temperatures, which merge at low temperatures close to *T*_g_. The time constants from HF and FC ^31^P NMR agree with those from DLS. Presumably, they characterize the overall reorientation of the molecule, i.e., of its PO_4_ core. The FC ^1^H NMR correlation times are somewhat shorter, which can be rationalized by additional the internal motions of the phenyl rings and their attached methyl groups carrying the interacting protons. Surprisingly, the dielectric spectra show a bimodal structure at high temperatures, while they exhibit a single peak close to *T*_g_ (see inset of Figure 5b). The time constants of the fast DS process are similar to those of ^1^H NMR, whereas the slow dielectric process has no NMR or DLS counterpart and its origin is still elusive. Possibly, it reflects the collective dynamics probed by dielectric cross correlations, as discussed for monohydroxy alcohols or glycerol (see Section 3.4).

The merging of the various rotational correlation times upon approaching *T*_g_ suggests that the distinct internal dynamics become slaved by the glassy dynamics, possibly because of the cooperativity of the α-relaxation in the highly viscous liquid. Phenomenologically, a similar behaviour was recently proposed for the non-rigid sorbitol molecule undergoing the glass transition [35]. There, site-specific NMR relaxation revealed different relaxation stretching at high temperatures, while displaying a similar trend at low temperatures. A recent study on sizable molecules to which a polar side group is attached showed a similar phenomenon [95].

In addition, we can see in Figure 5b that the self-diffusion coefficients *D*(*T*) obtained from FC ^1^H NMR relaxometry based on the low-frequency square root dispersion law (see Equation (5)) agree with those from static field gradient NMR [53].

### 3.2. Ionic Liquids

Thus far, we have focused on neat molecular glass formers. Ionic liquids, and especially room temperature ionic liquids (RTILs), are another class of liquids that are currently receiving a great deal of attention in view of their interesting properties and versatile applications [96,97,98,99,100,101]. Similar to conventional salts, such as NaCl, they consist only of anions and cations. However, a suitable choice of the ionic species allows one to lower the melting point or even fully suppress crystallization and convey good glass-forming ability [97,98,101,102]. The dynamics of RTILs resemble those of neat molecular glass formers in many respects [97,102,103,104]; however, the disparity and complexity of the ions also results in new features. In particular, many RTILs tend to decompose into nanoscopic polar and nonpolar domains, which may affect the dynamics [99,101,105,106,107].

**Figure 6:**
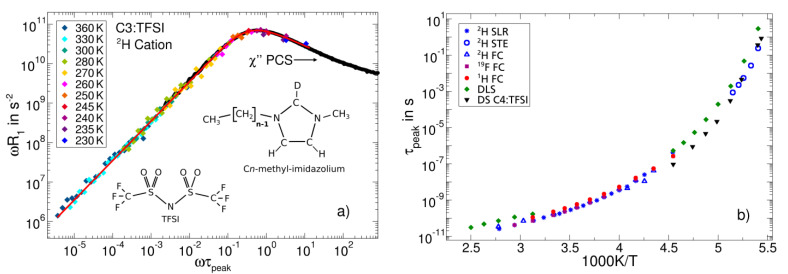
Reorientation dynamics of an ionic liquid comprised of 1-propyl-3-methyl-imidazolium cations (C3) and bis(trifluoromethylsulfonyl)imide (TFSI) anions: (**a**) ^2^H NMR susceptibility master curve for the selectively deuterated cation, constructed from FC and HF data at the indicated temperatures (colored symbols), together with the PCS susceptibility (black symbols) [111]. The red line is a CD fit of the NMR data. (**b**) Correlation times of cation reorientation from FC ^1^H and ^2^H relaxometry and from ^2^H stimulated-echo experiments (STE) [100], and of anion reorientation from FC ^19^F relaxometry together with data from DLS [111] and DS [112] studies, the latter for a slightly different ionic liquid consisting of 1-butyl-3-methyl-imidazolium cations (C4) and TFSI anions.

In Figure 6, we present the NMR susceptibility master curve of an exemplary RTIL, as obtained from FC and HF ^2^H spin-lattice relaxation data. Moreover, the chemical structures of the used cations and anions are depicted. Using an appropriately deuterated cation [100], ^2^H NMR allows us to selectively probe the single-particle reorientational dynamics of the imidazolium ring. However, FC ^2^H NMR studies are still challenging and rarely found in the literature [49,51,108,109,110]. The low gyromagnetic ratio leads to long experimental times and the strong quadrupolar coupling results in fast ^2^H spin-lattice relaxation, such that the susceptibility peak may be inaccessible by FC because of finite field-switching times. The latter drawback can be overcome when adding HF ^2^H relaxation data to extend the spectrum to higher frequencies, as is demonstrated in Figure 6a using results at a Larmor frequency of 46 MHz. The combination of FC and HF data clearly demonstrates the power of ^2^H relaxometry yielding a single-particle susceptibility, which probes the reorientation of specifically labelled molecular sites. The ^2^H NMR master curve agrees with PCS data [111] close to *T*_g_ when plotted over the reduced frequency axis $\omega \tau_{peak}$. Both susceptibilities show the α-relaxation peak and indications of a crossover to an excess wing at high frequencies. A CD fit limited to the relaxation peak reveals a width parameter of *β*_CD_ = 0.32, which is lower than the value of *β*_CD_ ~ 0.5 observed for many molecular liquids. Thus, a proposed generic shape of the α-relaxation of pure molecular liquids, if existent, cannot be extended to ionic liquids.

While FC ^1^H and ^2^H NMR inform us about the cation dynamics of the studied RTIL, FC ^19^F NMR enables selective studies of anion dynamics [99]. The correlation times obtained from the different NMR techniques are displayed in Figure 6b. Cation and anion dynamics occur essentially on the same time scale and show the non-Arrhenius temperature dependence typical of molecular glass formers. Moreover, all NMR data, including results from ^2^H stimulated-echo experiments at temperatures near *T*_g_, agree with the DS [112] and DLS correlation times [111]. Altogether, the dynamics of the studied ionic liquid exhibit only a moderate heterogeneity with a broadened, but still “CD-like”, susceptibility. However, the situation changes when cations with long alkyl chains are used. Then, structural inhomogeneities [107] give rise to more pronounced dynamical heterogeneities and a decoupling of cation and anion dynamics [99,101].

### 3.3. Salt Solutions

**Figure 7:**
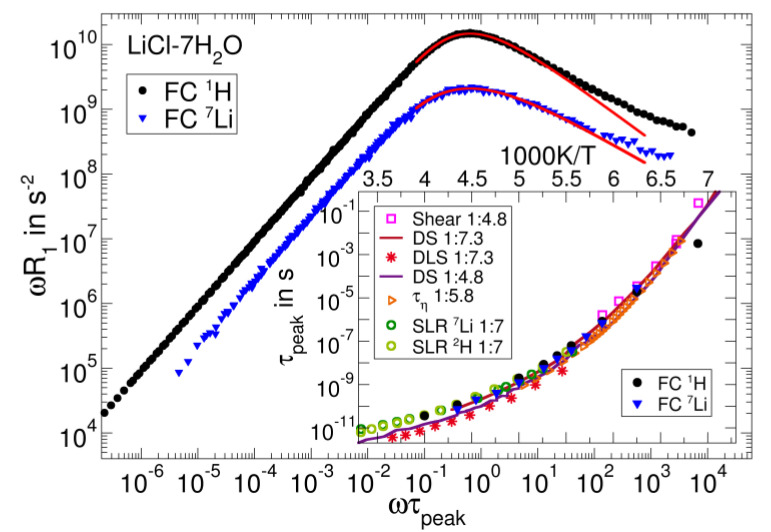
^1^H (black circles) and ^7^Li (blue triangles) FC master curves of LiCl-7H2O on a reduced frequency axis, which is defined by the respective peak correlation times *τ*_peak_ form KWW fits to the peak regions (red lines). The inset compares the FC peak correlation times with data from shear relaxation [119], DS [117], DLS [117], viscosity measurements [116], and conventional HF NMR [118] for the indicated aqueous salt solutions.

Likewise, aqueous salt solutions show a rich dynamical behaviour; in particular, the couplings of the ions with their hydration shells have received considerable scientific attention. A prominent example of such three-component systems is LiCl-7H_2_O solution, which can readily be supercooled [113,114,115]. Field-cycling NMR relaxation studies can employ the ^1^H (or ^2^H) and the ^7^Li (or ^6^Li) nucleus to monitor the dynamics of the water molecules and the dissolved lithium ions, respectively. Hence, the isotope selectivity of NMR again allows us to separately study the dynamics of the components, in contrast to, for example, DS. Figure 7 displays the FC ^1^H and ^7^Li NMR susceptibility master curves on a reduced frequency scale defined by the corresponding peak correlation times *τ*_peak_ at 180 K. When comparing these results, we need to consider the fact that the dominant interaction is different for ^1^H and ^7^Li. The ^1^H NMR results are dominated by the intramolecular magnetic dipole–dipole interaction between both the protons of the water molecule and, hence, report on water reorientation, whereas ^7^Li NMR probes the EFG at the lithium site, which fluctuates both when the lithium ion moves to a new environment and when the water molecules in its hydration shell rearrange. Therefore, the ^7^Li NMR susceptibility reflects a multi-particle correlation function, which involves local translational and rotational motions. The ^1^H NMR susceptibility shows the above discussed spectral shape with an α peak and an excess wing, indicating that the reorientation of the water molecules in the salt solution resembles that in neat molecular liquids. For ^7^Li, the accessible frequency range is more limited, but indications for an excess wing can still be observed.

A closer comparison shows that the peak of the ^1^H susceptibility is narrower than that of the ^7^Li susceptibility. Explicitly, KWW fits perform slightly better than CD fits in these cases and yield stretching parameters of *β*_K_ = 0.68 for ^1^H and *β*_K_ = 0.50 for ^7^Li. The inset shows the peak time constants derived from these fits and from the shift factors of the master curves. Despite their different microscopic interpretation, the ^1^H and ^7^Li NMR correlation times are very similar and agree with those obtained from the viscosity of LiCl-5.8H_2_O [116]. Moreover, they are in harmony with DS results [117] for LiCl-7.3H_2_O and HF ^2^H and ^7^Li NMR data [118] for LiCl-7D_2_O. Depolarized light scattering [117] probes slightly faster dynamics than the other methods. Altogether, the comparison reveals that the fluctuations of the ^1^H, ^2^H, and ^7^Li NMR interactions report on the structural relaxation of LiCl solution.

**Figure 8:**
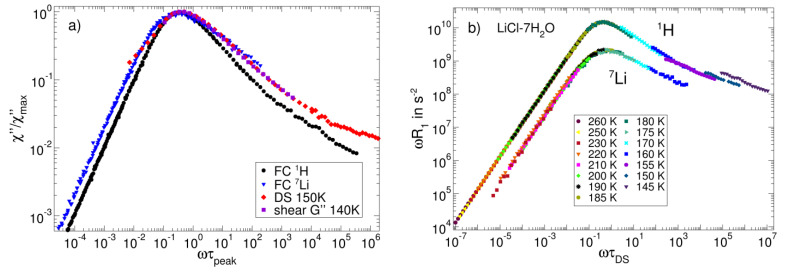
(**a**) Field-cycling ^1^H and ^7^Li susceptibilities compared to DS and shear relaxation susceptibilities at 150 K and 140 K, respectively, plotted over a reduced frequency scale $\omega \tau_{peak}$. The data are scaled by their maximum values. (**b**) $\omega R_{1}\left(T\right)$ ^1^H and ^7^Li FC data, rescaled horizontally by the respective $\tau_{DS}\left(T\right)$ from DS data [117]. Most FC data collapse onto a master curve. Deviations occur at low temperatures, where a secondary water process separates from the α-process [120].

The data collection in Figure 7 also reveals that the solutions with salt concentrations from LiCl-4.8H_2_O to LiCl-7.3H_2_O show similar dynamics, justifying a more detailed comparison of our data with results for samples of slightly different compositions. In Figure 8a, we see that the shape of the ^7^Li FC susceptibility is similar to that from shear relaxation and DS [119], whereas the ^1^H FC susceptibility peak is significantly narrower. As mentioned earlier, ^7^Li NMR probes the fluctuations of the local lithium ionic environments, thus reflecting both solvent and solute dynamics. Likewise, shear rheology and DS probe multi-particle dynamics associated with the local friction and the fluctuating electric dipoles of the charged and polar constituents, respectively. This common multi-particle origin may provide a rationale for the similar spectral signature of these susceptibilities. On the other hand, ^1^H FC relaxometry mainly probes the reorientation of individual water molecules, which may be a less diverse dynamical process, leading to a narrower susceptibility.

To further scrutinize whether FTS holds even in such complex three-component systems, we re-analysed the FC data by scaling the frequency axis with the DS correlation times $\tau_{DS}$ [117] (see Figure 8b). The ^1^H and ^7^Li data collapse onto a master curve for all temperatures above 160 K, indicating that NMR and BDS yield the same temperature dependence of the α-process and that FTS is valid. Furthermore, this scaling reproduces the different broadening of the ^1^H and ^7^Li susceptibilities found in Figure 7. In contrast, the construction of a ^1^H master curve fails below 160 K, implying that the water reorientation decouples from the α-relaxation near *T*_g_. Further analyses are required to decide whether or not this effect is related to the water-dominated secondary-ν relaxation appearing in various types of aqueous systems at sufficiently low temperatures [118,120].

### 3.4. Monohydroxy Alcohols

Monohydroxy alcohols show complex dielectric spectra exhibiting an additional relaxation—the so-called Debye relaxation—which is slower and usually stronger than the α-relaxation. There is widespread agreement that the Debye process is linked to the formation of hydrogen-bonded supramolecular structures [121]. For a long time, the Debye process was considered merely as a dielectric feature. However, recent rheology studies [121,122] have demonstrated that monohydroxy alcohols show slower-than-structural-relaxation dynamics, which are commonly considered specific to polymers.

In order to investigate a possible link between the slower-than-structural-relaxation dielectric and rheological processes and the separation of translational and rotational dynamics (in terms of the *r* value discussed in Section 3.1), we applied FC ^1^H NMR to a series of octanol isomers, namely 3-, 4-, 5-, and 6-methyl-3-heptanol, which were considered in the above mentioned rheological study [123]. The goal was to determine whether or not the position of the methyl group controlled the formation of the supra-molecular structures responsible for the Debye process. A comparison of the rheological and dielectric relaxation spectra suggested that the size of the supramolecular structures was unchanged, while the dielectric strength varied by a factor of 100 among this series of octanol isomers [123].

**Figure 9:**
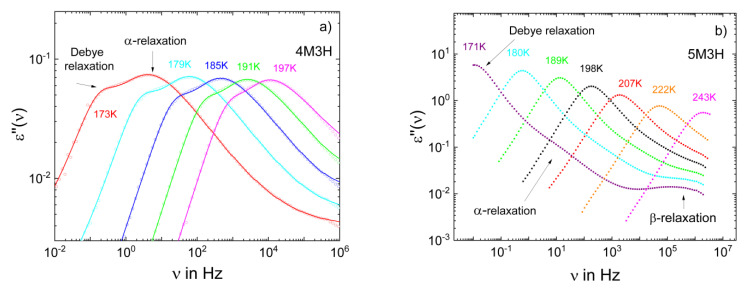
The dielectric spectra of (**a**) 4-methyl-3-heptanol (4M3H) and (**b**) 5-methyl-3-heptanol (5M3H). The spectra of 4M3H are fitted by a sum of a Debye and a Havriliak–Negami function.

**Figure 10:**
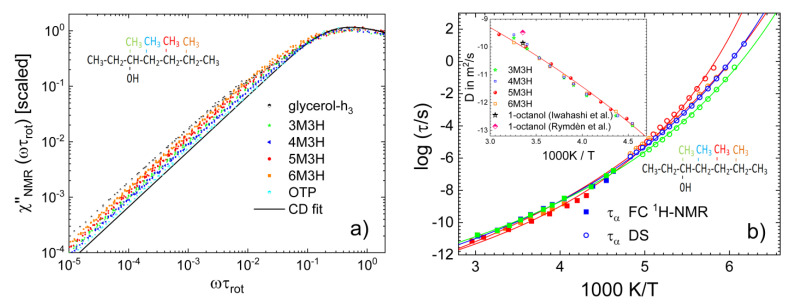
(**a**) The low-frequency part of the FC ^1^H NMR susceptibility master curves of the four octanol isomers. For comparison, the susceptibilities of glycerol-h_3_ and o-terphenyl and a CD susceptibility are included. (**b**) Rotational time constants obtained from DS (open symbols) and FC ^1^H relaxometry (solid symbols). Inset: diffusion coefficients determined by FC ^1^H relaxometry and from the literature [124,125]. The solid lines are guides for the eye.

Figure 9 presents the dielectric spectra of two of these isomers. Clearly, the relative strength of the Debye relaxation, as well as the overall relaxation strength, drastically changed between 4- and 5-methyl-3-heptanol, while the rheological measurements suggested that the size of the supramolecular structures was hardly altered [122]. In Figure 10a, we display the FC ^1^H NMR susceptibility master curves for the four octanol isomers together with those of o-terphenyl and glycerol. A low-frequency shoulder is clearly recognized for the monohydroxy alcohols. It is weaker than for glycerol, but certainly stronger than for o-terphenyl. Specifically, *r* values of 22–34 were extracted. The difference between the isomers was too small to suggest a trend for the influence of the methyl group position on the *r* value, while the dielectric strength of the Debye process strongly varied across this series [123]. Hence, there is no clear-cut relation between the *r* value and the manifestation of the Debye relaxation. Fits of the master curves yield *τ*_rot_(*T*) (see Section 2). In Figure 10b, we can see that the FC NMR results extend the DS data to higher temperatures and show that the position of the methyl group also does not significantly change the rotational correlation times of the octanol isomers.

**Figure 11:**
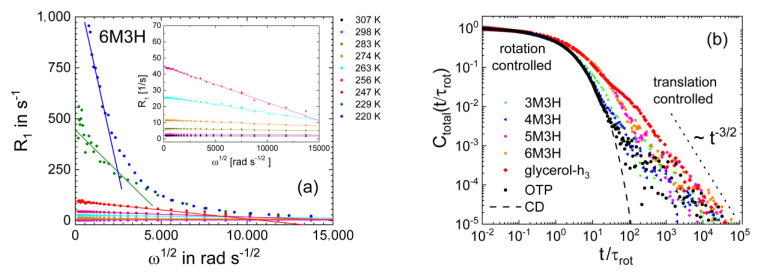
(**a**) ^1^H spin-lattice relaxation rate R_1_ as a function of the square root of the frequency, ω^1/2^. The self-diffusion coefficient *D* is determined from the linear part of *R*_1_(*ω*) via Equation (4), as indicated by the straight lines. Inset: magnification of the high-temperature data. (**b**) Normalized NMR time correlation functions derived from the susceptibility master curves of the octanol isomers (see Figure 9a), glycerol-h_3_, and o-terphenyl (OTP) in a double logarithmic representation. The dashed line is a CD function representing the rotational dynamics and the dotted line indicates a long-time *t*^−3/2^ power law reflecting the translational dynamics.

As discussed in Section 2, the analysis of the low-frequency FC ^1^H NMR data provides straightforward access to the self-diffusion coefficient *D*(*T*) using the universal low-frequency square root dispersion law Equation (4). The results for glycerol and m-TCP were already presented in Figure 3b and Figure 5b. The approach is illustrated in Figure 11a. Plotting *R*_1_(*ω*) as a function of *ω*^1/2^ and analysing the linear regime directly yields *D*. The diffusion coefficients resulting from this analysis for the octanol isomers are shown in the inset of Figure 10b. They agree with the data in the literature [123,124] and virtually do not depend on the position of the methyl group, as do the reorientational correlation times. Finally, the correlation functions obtained from the FC ^1^H NMR susceptibility master curves of the octanol isomers—more precisely, from the corresponding spectral densities—by Fourier transformation are displayed on a double logarithmic scale in Figure 11b. In this representation of the data, we clearly recognize the different separations of rotational and translational motions, which are reflected by the different *r* values. For all studied liquids, a decay due to rotational motion, which is described by the CD function, was followed by a decay due to translational motion, which exhibits the characteristic long-time t^−3/2^ power law of dipolarly coupled spins. For glycerol-h_3_ with a large *r* value of ~60, a bimodal decay was most pronounced.

### 3.5. Confined Liquids

Next, we move from bulk to confined liquids. It is well established that nanoscale geometrical restrictions have substantial effects on the thermodynamics, structures, and dynamics of liquids, which have important implications for biological, geological, and technological processes [126,127,128,129,130]. However, the influence of nanoscopic confinements on the shape of dynamical susceptibilities is still elusive. In large part, this lack of knowledge can be attributed to serious drawbacks of DLS and DS approaches to confined liquids. Specifically, many of the common host materials, e.g., mesoporous silica, are nontransparent powders, such that the enclosed liquids are inaccessible to optical experiments. Moreover, the structural heterogeneity of confinement samples causes internal polarization effects in dielectric studies, which often mask contributions from slow dynamical processes. On the other hand, NMR methods have proven to be very useful to ascertain not only the phase behaviors and structural properties of pure and mixed confined liquids, but also their translational and rotational motions [129,130,131,132]. Here, we show that FC ^1^H and ^2^H NMR provide valuable information about the dynamic susceptibilities of confined liquids.

**Figure 12:**
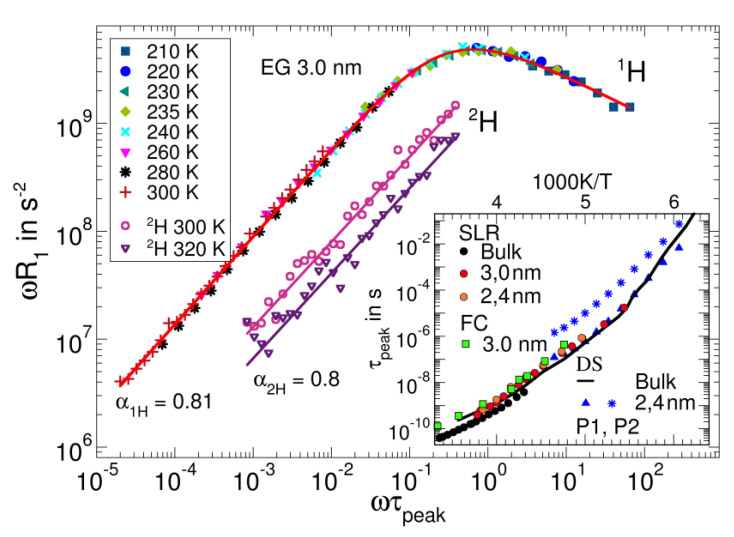
FC ^1^H and ^2^H NMR susceptibilities of ethylene glycol in cylindrical silica pores with a diameter of 3.0 nm. For ^1^H, the susceptibility master curve constructed by horizontally shifting the data sets at the indicated temperatures is shown together with a HN fit (red line), yielding the shape parameters α = 0.81 and β = 0.45. For ^2^H, the susceptibilities at 300 K and 320 K are interpolated with power laws ω^0.8^ and shifted horizontally for better visibility. The inset compares the correlation times obtained from the position of the ^1^H susceptibility peak at 235 K and the shift factors used for the construction of the master curve with correlation times from HF ^2^H relaxation and DS studies on ethylene glycol in various silica pores and in the bulk [137,138,142]. DS studies find two dynamic processes, P1 and P2, from which P1 corresponds to the main NMR (α) relaxation process.

In FC NMR approaches to confined liquids, it is a priori unclear whether FTS applies. In particular, MD simulation studies have reported that the magnitude and range of wall effects on the α-relaxation of confined molecular liquids increase upon cooling [133,134,135]. Here, we confined ethylene glycol to silica pores, which have a well-defined cylindrical shape and a pore diameter of 3.0 nm [136,137,138], allowing us to fully suppress crystallization [138]. Figure 12 shows the NMR susceptibility master curve constructed by shifting the FC ^1^H relaxometry data of ethylene glycol at temperatures in the range of 210–300 K along the frequency axis. Despite minor deviations in the low-frequency flank, which may result from the aforementioned temperature-dependent wall effects, the data collapse was satisfactory, indicating that possible FTS deviations were weak, at least for the studied system and temperatures. This was confirmed by the observation that the correlation times obtained from the shift factors together with the peak position at 235 K agreed with those from other NMR and DS studies on confined and bulk ethylene glycol [138,139] (see the inset of Figure 12).

The NMR susceptibility of confined ethylene glycol differs in an important aspect from that of the above bulk liquids—it does not exhibit a CD shape, but the slope of its low-frequency flank is notably smaller than +1. Explicitly, the master curve is well described by the Havriliak–Negami function, yielding ω^0.8^ for the low-frequency flank. This low-frequency broadening is not caused by the above-mentioned contributions of translational motion to the ^1^H spin-lattice relaxation. In particular, for the FC ^2^H susceptibilities—which are not governed by dipolar but rather by quadrupolar interactions and, hence, are free of these contributions—we also observe a ω^0.8^ behavior. Moreover, the ^2^H data confirm that the reduced low-frequency slope is not an artifact resulting from the construction of a master curve. On the other hand, for ^2^H, FC relaxometry alone does not provide access to the susceptibility maximum because the finite field-switching times of the setup do not allow for measurements of the short spin-lattice relaxation times in this region.

Altogether, we conjecture that the ethylene glycol dynamics in the pore center are bulk-like and dominate the peak region of the susceptibility, providing a rationale for the similar peak positions of the bulk and confined liquids (see the inset of Figure 12), whereas the dynamics near the pore wall are slowed, leading to a broader low-frequency flank for confined liquids than that for bulk. Thus, the NMR susceptibility indicates that liquids exhibit stronger dynamical heterogeneities in confinements than in bulk. However, for confined liquids, one may not expect a generic shape of the dynamical susceptibilities. Rather, the shape depends on the magnitude of the wall effect, which is established by the specific guest–host interactions and has a range relative to the confinement and molecule sizes, which determine the relevance of bulk-like dynamics in the pore center. Finally, it should be noted that such confinement effects are not restricted to liquids in porous materials; they may also occur in dynamically asymmetric mixtures, such as polymer and protein solutions, where the less mobile larger molecules at sufficiently high concentrations form confinements for the more mobile smaller molecules, which are soft or solid at temperatures above and below the glassy transition [30,140,141].

### 3.6. Field-Cycling NMR Experiments Addressing the β-Relaxation of Pure Molecular Liquids

**Figure 13:**
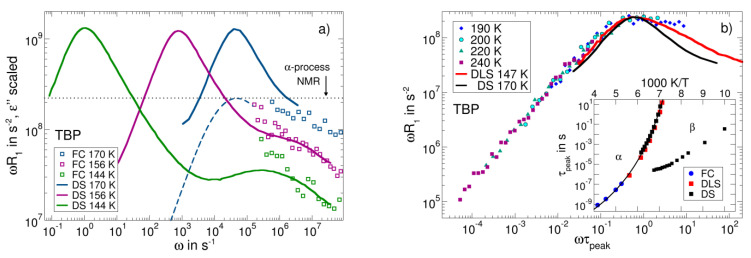
(**a**) Dielectric spectra (DS, solid lines) [143,144] corrected for the Curie factor and scaled in amplitude by a global factor to match the FC ^31^P NMR susceptibility (open symbols) of tributyl phosphate (TBP) at the indicated temperatures [53]. The blue dashed line is the interpolated NMR data from panel (**b**) re-scaled with $\tau_{\alpha }$(*T* = 170 K). (**b**) Susceptibility master curve of TBP constructed from FC ^31^P relaxation data at temperatures from 190 K to 240 K on a reduced frequency scale *ωτ*_peak_. A DS spectrum at 170 K [144] and a DLS spectrum at 147 K are included [27], both shifted to the NMR peak. Inset: correlation times of the α- and β-processes from FC ^31^P NMR relaxometry, DS [143], and DLS [27]. The black line is a guide for the eye.

The nature of the β-process is still incompletely understood. As revealed by solid-state NMR [55,56,57,58], the restricted reorientations of essentially all molecules are involved. Dielectric spectra characteristic of the situation above *T*_g_ are shown for the case of tri-butyl phosphate (TBP; *T*_g_= 140 K) in Figure 13a [143]. The β-relaxation is weaker than the α-relaxation but shows an increasing trend with temperature, a well-known feature of the β-relaxation above *T*_g_ [1]. Thus far, a direct comparison of DS and NMR susceptibilities covering both the α- and β-relaxation is not available. FC ^31^P NMR relaxometry appears promising to probe the α- and β-relaxations because the accessible frequency range is broader than in FC ^1^H relaxometry, which is a result of the fact that the heteronuclear ^31^P-^1^H dipolar couplings dominating the former nucleus are weaker than the homonuclear ^1^H-^1^H couplings of the latter [53].

Figure 13b shows the NMR susceptibility master curve constructed from the FC ^31^P relaxation data of TBP at 190–240 K [53]. It is dominated by the α-relaxation. Compared to the dielectric spectrum at a somewhat lower temperature of 170 K, the ^31^P NMR relaxation peak is much broader—in particular, on its high-frequency flank. The latter effect results most likely from the coalescence of the α-relaxation with the β-relaxation at the higher temperatures of the NMR measurements. The FC correlation times obtained from the peak position at 190 K and the shift factors from the master curve construction agree with the extrapolation of the DS [143] and DLS [27] data (see inset of Figure 13b). Thus, FC ^31^P NMR considerably expands the dynamic window of the former methods to faster molecular reorientation at higher temperatures. The DS time constants of the β-relaxation show a trend to merge with those of the α-relaxation at 180 K–190 K, corroborating the conjecture that the maxima of the NMR susceptibilities at these temperatures are broadened because the β-relaxation has not yet fully merged with the α-relaxation.

**Figure 14:**
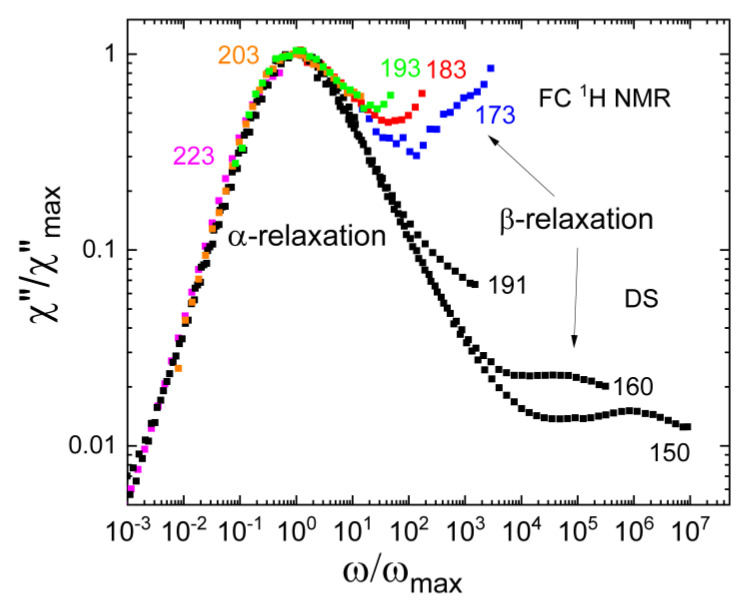
Susceptibility master curve of cyanocyclohexane from FC ^1^H NMR relaxometry together with dielectric spectra (black symbols) at the indicated temperatures in K [145]. The data are scaled for the best overlap of the α-relaxation peak. The FC and DS data show a secondary (β-) relaxation at high frequencies. However, the amplitude of the β-relaxation with respect to that of the α-relaxation is significantly higher in FC than in DS.

In Figure 13a the dielectric spectra were scaled to overlap with the FC data at high frequencies, i.e., in the region of the β-relaxation. The maximum value of the NMR susceptibility associated with the α-relaxation outside the accessible frequency range is determined by the NMR coupling constant. In Figure 13b, this expected height of the α- peak is indicated by the dashed horizontal line. We observe that the relaxation strength of the β-processes relative to this value of the α-process is much larger than that seen in the dielectric spectra. Thus, the results for TBP suggest that the relative relaxation strength of the β-relaxation is higher in FC than in DS. Similar results were reported for the plastic crystalline phase of cyanocyclohexane [145]. This is demonstrated in Figure 14, where the rescaled FC ^1^H NMR susceptibilities show a stronger β-relaxation than the corresponding dielectric spectra. Solid-state ^2^H NMR echo experiments on cyanocyclohexane have documented a very similar manifestation of the β-process to that in structural glasses, again suggesting highly restricted molecular wobbling motion [55,57]. Given that such hindered reorientation causes the β-process, one indeed expects that the susceptibility contribution of the β-relaxation is by a factor of three larger in NMR probing an *l* = 2 rotational correlation function than in DS observing an *l* = 1correlation function, provided collective effects can be ignored [146,147]. Based on these arguments, the relative amplitude of the β-relaxation in PCS, which also probes *l* = 2 data, should be comparable to that in NMR. However, inconsistent with this expectation, the PCS susceptibility of TBP [27] shows a comparably weak β-process. In any case, detailed comparisons of NMR, DLS, and DS susceptibilities should help to further improve our understanding of the molecular nature of the α- and β-processes in glass-forming liquids in future work.

## 4. Conclusions

NMR relaxometry—most notably, the FC technique—is a versatile tool to determine the dynamical susceptibilities of glass-forming liquids over broad frequency ranges. Based on the isotope selectivity of this method, the experimental approach can be designed to deliver a specific aspect of the system under study. In particular, NMR relaxometry allows one to selectively address the components of liquid mixtures. Furthermore, depending on the probe nucleus, it is possible to investigate translational and rotational motions and to assess single-particle or multi-particle correlation functions. For example, FC relaxation dispersions provide straightforward access to both translational self-diffusion coefficients and rotational correlation times, enabling stringent tests of the SED relation in a single measurement.

For pure molecular liquids, the NMR susceptibilities revealed the structural (α) relaxation and secondary (β) relaxations, complementing the results from other experimental methods. The Cole–Davidson-like shape of the α-relaxation is usually similar in NMR and DLS approaches, while the width parameter *β*_CD_ is different in the dielectric spectra of especially polar liquids, likely due to often neglected contributions from dielectric cross correlations between different molecules. Moreover, FC NMR relaxometry studies confirmed that the excess wing is a generic relaxation feature of liquids near *T*_g_. However, the amplitude of the excess wing generally differs between various experimental methods, likely because of their different sensitivities to small-amplitude motions [55]. This also holds for the relaxation strength of the β-relaxation. By using ^1^H as the probe nucleus and analysing the inter- and intramolecular contributions to the relaxation of the dipolarly coupled spin systems, it became clear that the time-scale separation between translational and rotational motions differs for various types of liquids. Specifically, translation is more retarded with respect to rotation for liquids with fully established hydrogen-bond networks than for van-der-Waals liquids, which virtually obey the SED prediction. On the other hand, the NMR susceptibilities of hydrogen-bonded liquids did not provide evidence that the extent of the translation-rotation separation is correlated with the magnitude of the Debye process, which is a slow relaxation that is commonly attributed to a reorganization of hydrogen-bonded supramolecular aggregates.

Concerning liquid mixtures, FC NMR approaches may use various probe nuclei to, for example, selectively assess the dynamics of specific anion and cation entities in ionic liquids or investigate the dynamical couplings between ions and their hydration shells in aqueous salt solutions. These possibilities reveal that the characteristic Cole–Davidson-like shape of the α-relaxation is retained in liquid mixtures, provided that structural or dynamical heterogeneities are moderate. Nevertheless, the width parameter *β*_CD_ may differ for the various components of an ionic liquid or a salt solution. Thus, there is no generic shape of the α-relaxation in liquid mixtures. In contrast to the findings for liquids in the bulk, for an exemplary liquid in nanoscopic confinement, we observed that the low-frequency flank of the α-relaxation had a slope smaller than unity, i.e., it was notably broadened, most likely due to a slowing of liquid dynamics near the pore wall. One may expect that a similar broadening occurs in dynamically asymmetric liquid mixtures, where the slower component forms an intrinsic confinement for the faster one.

Although the use of ^1^H FC NMR relaxometry was reported in pioneering works several decades ago, the capabilities of the method have been largely overlooked for years and have only recently begun to be fully explored. In particular, FC studies using other nuclei, such as ^2^H, ^7^Li, or ^31^P, are still rare; however, they have the potential to provide detailed insights into the dynamics of glass-forming pure and mixed liquids. Moreover, it appears to be a fruitful strategy to apply FC NMR relaxometry to other types of condensed matter systems in future work. For instance, a promising route may be the application to dynamical couplings in biological and other soft matter. Furthermore, first examples [59,60,148] demonstrate the suitability of FC NMR relaxometry for the characterization of ion dynamics in materials for energy storage and conversion, including solids for future battery technologies.

## Data Availability

The data that support the findings of this study are available from the corresponding author upon reasonable request.
